# Obsessive–compulsive disorder—contamination fears, features, and treatment: novel smartphone therapies in light of global mental health and pandemics (COVID-19)

**DOI:** 10.1017/S1092852920001947

**Published:** 2020-10-21

**Authors:** Baland Jalal, Samuel R. Chamberlain, Trevor W. Robbins, Barbara J. Sahakian

**Affiliations:** 1 Department of Psychiatry, University of Cambridge School of Clinical Medicine, Cambridge, United Kingdom; 2 Behavioural and Clinical Neuroscience Institute, University of Cambridge, Cambridge, United Kingdom; 3 Department of Psychiatry, Faculty of Medicine, University of Southampton; and Southern Health NHS Foundation Trust, Cambridgeshire & Peterborough NHS Foundation Trust, United Kingdom; 4 Department of Psychology, University of Cambridge, Cambridge, United Kingdom

**Keywords:** Obsessive–compulsive disorder, contamination fears, smartphone therapies, global mental health, COVID-19.

## Abstract

This review aims to shed light on the symptoms of obsessive–compulsive disorder (OCD) with a focus on contamination fears. In addition, we will briefly review the current therapies for OCD and detail what their limitations are. A key focus will be on discussing how smartphone solutions may provide approaches to novel treatments, especially when considering global mental health and the challenges imposed by rural environments and limited resources; as well as restrictions imposed by world-wide pandemics such as COVID-19. In brief, research that questions this review will seek to address include: (1) What are the symptoms of contamination-related OCD? (2) How effective are current OCD therapies and what are their limitations? (3) How can novel technologies help mitigate challenges imposed by global mental health and pandemics/COVID-19.

## Introduction

Obsessive–compulsive disorder (OCD)—characterized by obsessions and/or compulsions—is a deeply enigmatic neuropsychiatric disorder. Once thought rare, OCD is now considered one of the most common psychiatric conditions, afflicting 2% to 3% of the general population.^1-3^ This condition is debilitating, associated with immense suffering worldwide, and costly.^4^ ^,^^5^ Indeed, disorders of the brain including OCD have a yearly cost of €134 billion in the United Kingdom alone^6^ (on the cost of mental illness see also Sahakian^7^). In the United States, the annual cost of OCD is estimated at $10.6 billion.^4^ Unsurprisingly, therefore, mental conditions like OCD, and neurological and substance-use disorder combined, comprise 13% of the global disease burden, greater than the burden of diseases such as cancer and cardiovascular disorder^8^ (see Hollander et al^9^).

One of the most common and striking types of OCD, affecting up to 46% of patients, is characterized by severe contamination fears and excessive washing behaviors.^10^ ^,^^11^ These patients can experience great distress even when touching, seemingly harmless, objects such as a doorknob and may engage in excessive cleansing behaviors for hours, resulting in skin irritation or bleeding.^12^ In some cases, these exasperating cleansing routines result in patients being unable to leave their home (eg, Cyr^13^).

Despite the distressing nature and great economic burden of OCD, the existing treatments have limitations. This review aims to shed light on the symptoms of contamination-related OCD. In addition, we will briefly review the current therapies for OCD and detail what their limitations are. A key focus will be on discussing how technological solutions may provide approaches to novel treatments, especially when considering global mental health challenges (see Stein^14^) and restrictions imposed by global pandemics such as COVID-19. In sum, research questions this review will seek to address include: (1) What are the symptoms of contamination-related OCD? (2) How effective are conventional OCD therapies and what are their limitations? and (3) How can novel technologies help overcome challenges imposed by global mental health and pandemics/COVID-19.

## The Role of Disgust in OCD

Research suggests that there is an association between OCD symptoms including contamination fears and disgust. ^15^ ^,^^16^ It has specifically been shown that disgust sensitivity and anxiety are two independent factors driving contamination fears; indeed, disgust sensitivity predicts contamination fears after controlling for trait anxiety and anxiety sensitivity^17^ (for a theoretical model on disgust vis-à-vis contamination fears see Olatunji et al^18^). Relatedly, research has shown that individuals with contamination fears have difficulties disengaging from both fear and disgust stimuli.^19^ This is consistent with the finding that disgust sensitivity is associated with attention bias toward disgust stimuli.^20^ Moreover, aberrant disgust processing in the brain may contribute to the pathophysiology of OCD^21^; in particular, the insula cortex.^22^ ^,^^23^ Elevated activation of the insula has been described in OCD.^24-26^ (on neural correlates of disgust in contamination-related OCD, see Lawrence^27^).

Cognitive errors may contribute to disgust reactivity in OCD. One example is *sympathetic magic*, which involves irrational ideas regarding the spread of contagion; that contamination can spread in a rapid and dynamic fashion, across a chain of objects many degrees removed from the initial contaminated object.^23^ ^,^^28^ (for an investigation in OCD, see Tolin et al^29^; on the related concept of “looming vulnerability,” see Riskind et al^30^). The “law of contagion” and “law of similarity” are thought to underlie sympathetic magic beliefs. According to the law of contagion once an object has come in contact with a contaminated item it will always remain so; exemplified by reluctance to drink a cup of coffee that has been stirred with a used but washed spoon.^23^ ^,^^28^ ^,^^29^ The law of similarity dictates that an item’s visual resemblance to a contaminated one is enough for it to also become contaminated.^23^ For example, healthy volunteers are hesitant to eat chocolate shaped as feces, even when told that the objects are food items.^28^ ^,^^31^ ^,^^32^

Notably, research suggests that disgust reactions in individuals with clinical contamination fears (OCD) are amenable to exposure therapy.^33^ In fact, OCD patients are likely to experience overall symptom improvement, if treatments reduce disgust propensity.^22^ (see also Athey et al^34^). By comparison, while acute administration of the selective serotonin reuptake inhibitor (SSRI) escitalopram results in greater disgust accuracy and sensitivity in OCD, disgust hyper-responsivity does not normalize following chronic SSRI treatment.^35^ Taken together, developing novel exposure-like treatments that target disgust systems in OCD represents a promising avenue for future research.^28^

## A Bayesian Computational Model

A novel model grounded in a Bayesian brain framework suggests that OCD is characterized by certain computational deficits^36^ (for a conceptually related model, see Szechtman and Woody^37^). Specifically, it posits that OCD patients have difficulties in depending on past events when predicting the effects of their own actions and future possible events. In Bayesian terms, this is referred to as *excessive uncertainty regarding state transitions*. Put simply, within the Bayesian framework, the brain makes inferences (ie, probabilistically) about the actual state of the world which is unobservable.^38^ ^,^^39^ It does so by making predictions; that is, based on prior knowledge (top-down) and integrates this information with incoming sensory input (bottom-up) (on this topic, see Teufel and Fletcher^40^). The level of certainty associated with each source of information (“noise”), determines their relative weight when making such inferences.^39^ When incoming sensory feedback is less noisy (certain), it is more informative.^36^

Fradkin et al^36^ propose that OCD patients tend *not* to rely heavily on past events when making inferences about the world, including their own actions, but instead over-rely on sensory input even in stable scenarios. Such *state uncertainty* deficits in OCD applies readily to compulsive washing. For example, after touching a doorknob, there is no sensory input to confirm that your hands are clear of contagion. Yet OCD-free individuals usually infer based on past knowledge (ie, about cause and consequence) that washing results in a contamination-free state. But for OCD patients, such probability of state transitioning is less certain leading to overreliance on sensory confirmation (unattainable in this case), and effectively, repeated handwashing. Elevated *transition uncertainty* in OCD, including sensory overreliance and overinterpretation may account for perfectionism propensities (eg, washing behavior being “not just right”^41^ ^,^^42^). The model also accounts for excessive habits.^43^ ^,^^44^ Given goal-directed behaviors are computationally costly in a world of heightened transition uncertainty (less predictability/controllability) resulting in repetitive behaviors, the brain defaults to (compensatory) habits. Notably, the face validity of this model was shown using quantitative computational simulations vis-à-vis existing empirical data. Finally, from this Bayesian perspective treatments like SSRIs and exposure therapy might exert their effects, in part, by reducing fear associated with elevated transition uncertainty.^36^

## OCD Treatment: An Overview

While the current paper focuses on contamination-related OCD, we consider treatments for OCD in general. This is because evidence-based treatments for OCD subtypes are lacking, and instead tend to target the disorder overall. Nonetheless, treatments for OCD in general are relevant for contamination fears, as this subtype is very common across the disorder, afflicting nearly half of all OCD patients.^10^

There are currently effective treatments for OCD—including the contamination subtype—but they have notable limitations and do not suit all patients. Indeed, up to 40% of OCD patients do not experience adequate symptom relief from treatment with first-line interventions.^45^ ^,^^46^ Moreover, data suggest that around 60% of OCD patients in the general community remain untreated (ie, based on a review of epidemiology studies^47^ ^,^^48^); and that patients on average initiate effective treatments 17 years after the onset of the disorder.^49^

The first-line psychological treatment for OCD is cognitive behavioral therapy (CBT), including exposure and response prevention (ERP), as recommended by the National Institute for Health and Care Excellence.^50^ ^,^^51^ ERP involves exposing patients to symptom relevant anxiogenic situations (eg, touching a toilet seat). The patient is then prevented from carrying out the neutralizing safety-behavior (eg, excessive cleansing) which eventually leads to habituation.^12^ ^,^^52^ ^,^^53^ Meta-analyses have found ERP to be effective for OCD; compared to control conditions, such as wait list, progressive muscle relaxation, and anxiety management training).^54^ ^,^^55^

ERP has notable limitations^56^ with some patients finding that they cannot tolerate it. As many as half of all patients who initiate treatment do not improve and only a quarter of patients are asymptomatic post treatment. Likewise, 20% cannot continue treatment (ie, drop out) and a quarter are unwilling to start ERP,^41^ ^,^^56-58^ largely because of fear of the procedures.^12^ ^,^^59^ Furthermore, high levels of anxiety during ERP for some patients may prevent sufficient habituation during treatment. Indeed, high levels of fear may obstruct emotional processing and thus impair learning which is why moderate fear activation might be more ideal therapeutically.^60^ ^,^^61^

Restricted accessibility is another key limitation of CBT; complicated by the fact that ERP might require many hours for improvement. For instance, CBT is commonly administered in a single weekly session, that is, for 12 to 14 weeks (1-2 hours per session).^62^ This may be costly for some patients and time-consuming for patients and therapists.^63^ Unsurprisingly, therefore, many patients treated with CBT do not receive adequate amounts.^64^ Limited accessibility—high cost, being time-consuming, inconvenience of delivery (eg, participant travel), and geographical isolation (eg, impacting rural areas)—is thus a major potential drawback of ERP.^48^ ^,^^63^ ^,^^65^ ^,^^66^

In terms of pharmacological intervention, SSRIs and clomipramine show efficacy vs placebo control; as confirmed by a large recent network meta-analysis^67^ (see Harris et al^66^). This network meta-analysis also reported that the efficacy of SSRIs and clomipramine does not differ significantly, and that the efficacy of individual SSRI drugs appeared to be comparable. It should be noted that this network meta-analysis has a number of limitations and that head-to-heat comparator clinical trials of active treatments were generally lacking. But given that SSRIs are typically associated with milder side-effects relative to clomipramine, SSRIs, are considered the first-line pharmacological treatment for OCD.^50^ ^,^^66-68^ Although some clinics provide combined pharmacological (SSRI or clomipramine) and psychological treatment (CBT), there is no clear evidence to conclude that combined treatment is superior to either drugs or CBT alone.^67^ ^,^^68^ However, SSRI monotherapy has been reported to be more cost-effective than either CBT alone or combined SSRI + CBT.^45^ When OCD patients are unresponsive to treatment, antipsychotic agents that block dopamine receptor activity can be added to serotonin reuptake inhibitors (SRIs) (eg, to reduce stereotyped behavior); as found to be effective according to meta-analyses albeit in relatively small numbers of studies, and not for all types of antipsychotic medications^69^ (see Fineberg^70^). In cases of extremely debilitating and refractory OCD, neurosurgery interventions (associated with risks due to their invasive nature) are sometimes used. These include procedures such as cingulotomy, anterior capsulotomy, and deep brain stimulation (DBS).^66^ ^,^^71^ ^,^^72^ Despite the favorable effects of pharmacotherapy, not all OCD patients benefit. According to some estimates, around 40% to 60% of the patients improve following SRI intervention.^73^ Moreover, one potential drawback of pharmacological treatment is the undesired side effects.^74^ ^,^^75^

To improve upon ERP, cognitive elements have been added to the treatment or been applied as a separate “cognitive therapy” modality^55^; an approach that entails targeting cognitive errors.^76^ One meta-analysis examined the effectiveness of ERP, cognitive therapy and their combination: Abramowitz et al^54^ reported mean effect sizes for ERP (*d* = 1.50), cognitive therapy (*d* = 1.19), and combined ERP and cognitive therapy (*d* = 0.99); that is, revealing a larger effect size for ERP (relative to control conditions, such as wait list, progressive muscle relaxation, and anxiety management training), than either cognitive therapy or their combination; yet suggesting benefits across all three approaches.^55^ ^,^^77^ Furthermore, this meta-analysis indicated that ERP led to greater reductions in OCD symptomatology than did cognitive therapy or ERP + cognitive therapy. Overall, these results are in line with the view that ERP should constitute the psychological treatment of choice for OCD.^77^ They also dovetail with other meta-analyses showing that more behavioral focused therapies for OCD tend to have greater efficacy than those emphasizing cognitive aspects.^78^ However, despite the efficacy of behavioral therapy vs the utilized control conditions, it has various limitations, as discussed previously.

## Remotely Technology-Delivered CBT

To address barriers to traditional treatment (improve accessibility) research has explored remotely delivered CBT.^48^ ^,^^65^ ^,^^66^ ^,^^79^ These include video-conference administered CBT (vCBT), where treatment is provided through a video-conference call, as an analog to in-person CBT; and telephone-delivered CBT (tCBT), similar to vCBT, except the patient and clinician cannot see each other. These CBT applications are delivered in real-time and usually require comparable clinician–patient interaction as in-person treatment.^48^ Controlled trials support the effectiveness of both vCBT^80^ and tCBT.^81^

Other remotely delivered CBT methods include computerized CBT (cCBT) and internet-based CBT (iCBT). These may involve reading modules about the rationale of CBT and receiving instructions to conduct *in vivo* exposure on a computerized device either offline (cCBT) or online (iCBT).^48^ ^,^^82^ One iCBT intervention (10 weeks), provided by Andersson et al^82^ included elements such as psychoeducation, cognitive restructuring, constructing an exposure hierarchy, and instructions to do *in vivo* ERP. In this study, iCBT was effective, yielding greater improvements in OCD symptoms than the control intervention (internet-based nondirective supportive treatment); reporting a large (between-group) effect size (*d* = 1.12).

Taken together, OCD patients appear open to incorporating technology-based intervention into their daily lives. One recent meta-analysis found that remote interventions for OCD are more efficacious than control (ie, attentional control group or wait list) and as effective as in-person CBT.^48^ Although promising in terms of widening the reach of OCD intervention, remote-CBT applications have limitations. For instance, computers, such as laptops, are not fully transportable, and may not be fully secure in terms of preventing external access to private information. They do not always allow for easy and instant access to treatment as patients go about their daily lives (ie, in places where symptoms naturally arise); for example, the gym, grocery store, park, or the bus or train.^65^

## Smartphone Interventions for OCD

The rise in smartphone technology offers an exciting new avenue for overcoming challenges of existing OCD therapies. Indeed, smartphone treatments lend themselves to “technology-based personalized medicine.”^83^ ^,^^84^ Smartphone solutions can be personalized for each patient, allowing for targeted therapies and for patients to partake in their recovery process, and promote the learning of adaptive strategies to eradicate compulsive urges.^84^ ^,^^85^ They can give patients direct feedback about their treatment progress, provide insight about their condition, as well as pave the way for clinicians to monitor patients’ progress in real-time and intervene swiftly if necessary.^86^ ^,^^87^

Smartphones can potentially make therapy more available to members of lower socioeconomic status communities and developing countries with insufficient access to mental healthcare.^8^ Smartphone technology is now adopted by most members of society across diverse social strata and wide age groups, including preadolescents and the elderly (ie, in the United States; see Pew Research Center^88^). According to one report, there were 3.9 billion smartphone subscribers globally in 2016; the overall number of smartphone subscribers is expected to rise dramatically by the year 2022.^84^ ^,^^89^ All in all, smartphones may allow healthcare systems to provide simple and low-cost solutions for treating OCD, which might facilitate higher treatment uptake, lower drop-out, and early intervention. Indeed, as this condition afflicts up to 2% to 3% of the general population^2^ ^,^^3^ with economic costs as noted estimated at $10.6 billion per year in the United States alone,^4^ and they may have significant public health and societal impact.

Despite such widespread smartphone use, few apps have been developed for treating OCD.^65^ That is, available apps include CBT-type interventions with limited empirical support.^90^ For example, the Mayo Clinic Anxiety Coach for anxiety disorders and OCD entails components such as psychoeducation, construction of fear hierarchies, progress tracking, and guidance to conduct exposure exercises^90^ ^,^^91^ (see Abramowitz et al^79^). Case reports suggest this app has promise, showing initial evidence of overall acceptability for children with OCD.^91^ ^,^^92^ Another example is “LiveOCDFree,” a self-help app-guided ERP treatment for OCD. This app provides guidance on ERP and includes specific components, such as help designing an exposure schedule, setting up an ERP hierarchy and reminders for ERP exercises.^65^ One open trial (noncontrolled) provided preliminary data in support of its efficacy and acceptability; the first study to assess the efficacy of a smartphone intervention for OCD. The study found the app (ie, 12-week intervention) improved OCD and anxiety symptomatology.^65^ A related approach relied on an integrated treatment model. It consisted of in-person ERP (3-5 sessions 90 minutes per session) and weekly phone calls, combined with an app (“nOCD”) which helped with ERP strategies; for example, customizing fear hierarchies, setting up schedules, and reminders to aid with ERP.^93^ An open pilot trial (noncontrolled) showed that 8 weeks of this integrated model resulted in a significant reduction in OCD symptoms. However, only around half of the patients utilized the app on a weekly basis and often for less than an hour, indicating that many did not find it useful. Finally, a cognitive training app (“GGRO”) was recently developed aiming to address OCD-related cognition/beliefs. App exercises include being presented with positive self-statements like “I take things as they come” and maladaptive ones like “Everything can end in a catastrophe.” Participants either accept these by pulling them toward themselves on the screen (ie, downwards) or reject them by throwing them away from themselves (ie, upwards). Research in (nonclinical) student samples, including a noncontrolled open trial and a randomized trial (crossover design) with a waitlist control group^94^ ^,^^95^ provided preliminary evidence in support of its use (2 or 3 minutes per day for 15 days) for reducing OCD-related beliefs and symptoms. All in all, limited empirical research is available on app-based interventions for OCD. This research is largely based on case reports, controlled student samples, and pilot studies in OCD without control conditions rendering such data preliminary. Nonetheless, early findings are promising, and suggest that more rigorous clinical trials would be valuable (for a summary of smartphone interventions for OCD see Table 1).

**Table 1.** Summary of Smartphone Interventions for OCD.

Name	Modality	Overview	Evidence
Contamination fear free	Novel/nontraditional approach	Watching video of one’s own handwashing, or repeatedly touching a contaminant	One controlled study (1 wk) in subclinical sample; Jalal et al^84^
GGRO	Cognitive therapy	Tasks to address OCD-related cognition/beliefs	One noncontrolled open trial, and one randomized trial (crossover design) with waitlist control group; 15 d and student samples; Roncero et al^94^ ^,^^95^
LiveOCDFree	CBT	Guidance on ERP, designing exposure schedules, setting up ERP hierarchies, and ERP reminders	One noncontrolled open pilot trial (12-wk); Boisseau et al^65^
Mayo Clinic Anxiety Coach	CBT	Psychoeducation, construction of fear hierarchies, progress tracking, and guidance on exposure exercises	Case reports; Whiteside et al^91^ ^,^^92^
nOCD	CBT	Customizing fear hierarchies, setting up schedules, and ERP reminders	One non-controlled open pilot trial (8 wk); Gershkovich et al^93^

Abbreviations: CBT, cognitive behavioral therapy; OCD, obsessive–compulsive disorder.

## Smartphone Interventions: “Vicarious Exposure”

We recently conducted research that may inform smartphone treatments for OCD.^96^ Interestingly, college students with OCD symptoms experienced disgust from simply observing an experimenter contaminating himself (touching a disgust-inducing object). Moreover, once the participants had become contaminated, they reported relief when observing the experimenter engaging in handwashing. We call this method of inducing contamination and relief: “vicarious exposure.” Based on this principle, our group tested two novel smartphone interventions for OCD (we call “contamination fear free”). Individuals with contamination fears either (1) watched a video recording of themselves washing hands, (2) repeatedly touching fake feces, or (3) doing arbitrary hand gestures (control procedure) on a smartphone four times a day, for a week. Notably, the two smartphone interventions but not the control condition were associated with significant improvements in set shifting and symptoms of OCD in this subclinical sample but did not impact mood.^84^ That cognitive flexibility improved is promising because impaired set shifting is thought to reflect repetitive and stereotyped symptoms of OCD,^97^ such as compulsive cleansing. (Interestingly, anteromedial subthalamic nucleus DBS in OCD was previously shown to be associated with improved cognitive flexibility.^72^ It is possible, but has yet to be demonstrated, that these treatment-related improvements in cognitive flexibility seen in the vicarious exposure study could stem from modulation of such brain regions.) Finally, we found high levels of adherence to the interventions, stressing their possible value for future evaluation in clinical populations. It would also be valuable to measure the neurobiological underpinnings of the cognitive and symptom improvement observed with the app.

These findings are congruent with research demonstrating that incorporating safety behaviors (ie, neutralizing compulsive behaviors) into treatments do not invariably impede treatment benefits and in fact can be therapeutically useful; for example, by making treatments more acceptable (eg, Levy and Radomsky^98^). The usefulness of adding safety behavior (such as cleansing acts) to exposure interventions has been shown in nonclinical groups with contamination concerns^99^ (see also van den Hout et al^100^) and OCD patients with contamination fears.^101^ All in all, such studies challenge the widespread notion derived from traditional learning models that utilizing safety behaviors is always counterproductive.^100^ Safety behaviors during treatment might lead to increased control and a more relaxed (less-agitated) state, resulting in increased willingness to confront contaminants and reduction in fear and avoidance behavior^101^ ^,^^102^ (see also Jalal et al^32^).

Smartphone solutions, such as outlined above, may help to overcome barriers of conventional treatment, including intolerability issues and limited accessibility (eg, due to geographical isolation) and sociomedical costs. The transportable nature of the interventions makes them suitable for minimally resourced settings, such as rural environments and low-income countries with restricted access to healthcare (eg, specialized treatments) (on this, see Jalal et al^12^). They can be tailored for individual patients (“personalized medicine”), allowing for targeted therapies that encourage patients to actively partake in their recovery process. Moreover, these interventions can be applied in many real-life environments where OCD symptoms occur (eg, at work, when dinning at restaurants, and commuting using public transportation). They can be rendered context-specific (potentially adding to ecological validity) which is therapeutically beneficial vis-à-vis extinction, allowing for learning to potentially generalize to real-world settings, where the contamination fear is initially acquired.^103^ ^,^^104^ Indeed, while vicarious exposure bears some resemblance to other indirect approaches, such as virtual reality–based exposure and imaginal exposure the latter are conducted in non-naturalistic environments (eg, the clinic and at home). Extinction may not fully apply to these artificial contexts, given limited stimulus generalization.^103^ Finally, they are fitting for modern society—with several billion smartphone subscribers globally (as noted, expected to rise massively by 2022 ^89^).^84^ ^,^^89^

## Contamination Fears and Treatment in Light of Global Pandemics (COVID-19)

Global pandemics like COVID-19 may represent a substantial burden to patients with contamination-related OCD. Indeed, while the impact of the current pandemic on OCD is unclear and could take weeks/months to become fully apparent, there is a great risk that it could exacerbate symptoms for some patients^105^; and lead to relapse in some patients in remission (eg, Kumar and Somani^106^). In this respect, one preliminary naturalistic study (the first to explore the impact of the COVID pandemic on OCD) examined changes in OCD symptoms as a result of the pandemic (ie, symptoms assessed before the lockdown and 6 weeks after the full quarantine began) in OCD patients.^107^ Notably, the study found an elevation in the severity of obsessions and compulsions after the COVID-19 quarantine started. In particular, having contamination symptoms prequarantine was linked to elevated OCD symptom exacerbation during the quarantine. OCD symptom remission status prequarantine was also linked with greater worsening of OCD symptoms during the quarantine. Taken together, this report suggests that the pandemic may lead to a significant worsening of OCD symptoms, especially in patients with contamination symptoms and those with a remission status before the quarantine began.

There are several mechanisms by which the pandemic may worsen OCD. Virus-related fears (coupled with financial stressors like job insecurity and social isolation) may worsen stress/anxiety and further facilitate inflexible habits,^108^ and effectively compulsive cleansing behaviors.^44^ Moreover, some OCD patients might be susceptible to a conditioned (contamination) fear-response to COVID, persisting beyond the pandemic. That is, research has demonstrated that OCD patients have difficulties shaking off fear responses when no longer threatening (exhibit impaired safety signalling).^109^ ^,^^110^

Notably, guidelines (eg, by the World Health Organization [WHO] and Center for Disease Control and Prevention [CDC]) and heightened media focus on hygiene aimed at reducing the spread of COVID-19 could feed into pre-existing clinical obsessions about contagion^105^ ^,^^110^; especially when fears and doubts are driven by OCD-related health anxiety.^111^ Curiously, during the pandemic, members of society are encouraged to act—vis-à-vis cleanliness—in a manner that a few months ago would have been considered “OCD-like.”^105^ Excessive societal focus on contamination and normalization of decontamination rituals (compounded by observing OCD-free family members engaging in elaborate cleansing behaviors) may reinforce and even justify maladaptive cognitions (eg, “germs are everywhere and inherently dangerous”). In turn, this could challenge the validity of common treatments like cognitive therapy that aim to help patients develop adaptive cognitive strategies to overcome such irrational thoughts. There are already reports of patients expressing to therapists that given how everyone behaves in an OCD-like manner, they had been correct “all along” (see Fineberg et al^110^). Relatedly, as reviewed, a key feature of ERP is that it paves the way for fear disconfirmation. Patients are exposed to “contaminants” to only realize later that this did not materialize into a catastrophic outcome (eg, illness)^112^; thus, decoupling stimulus–response links underpinning compulsions. Paradoxically, in the context of COVID-19 people are discouraged from exposing themselves to daily-life situations/objects that could serve as fear disconformity events (stimulus–response degradation). Fittingly, it was recently suggested in order to ameliorate distress (eg, clinical contamination fears) associated with public health messaging that these improve their clarity. To reduce uncertainty and paranoia, they should increase the public’s confidence as to which health behaviors are necessary.^113^

The COVID crisis highlights the potential usefulness of smartphone interventions for tackling OCD. Indeed, they may provide a much-needed platform for therapy during pandemic situations. Given restrictions on physical proximity, a proportion of mental health treatment is relocated online mostly in the format of telephone or videocalls (eg, Skype, FaceTime, and Zoom^110^). As reviewed, OCD patients appear to be accepting of such telemedicine which is encouraging,^48^ but it has limitations; for example, in terms of transportability (lack instant/easy access). Critically, pandemics are anticipated to result in an increase in mental health problems (eg, anxiety, depression, and OCD), which puts an unusually high demand on therapists’ time (leading to work stress and burnout), and can be costly for patients and society.^113^ This problem is further complicated by the fact that in some locations, clinical staff are unavailable to provide psychotherapy as they are urgently recruited to provide clinical aid to COVID-19 victims.^110^ Mental health issues are most likely to affect vulnerable populations; for example, lower-income groups with restricted access to healthcare (lack medical insurance, etc.).^113^ As discussed, smartphone therapies may overcome these challenges, and thus possibly help mitigate the substantial personal and sociomedical costs resulting from pandemics.

Smartphone interventions (eg, indirect exposure procedures) might offer a more tolerable treatment-route than conventional psychological therapies like ERP during pandemics. Given the distress associated with ERP (direct confrontation with contaminants), it may lead to further emotional instability and symptom exacerbation. Notably, a report was recently released by a working-group of international experts, guiding clinicians on how to manage OCD during COVID-19.^110^ Indeed, one of their recommendations is that pharmacological intervention be the treatment of choice for contamination-related OCD. It was suggested that clinicians should consider pausing ERP (or adapting it). This was suggested in light of risks linked to ERP, like the confusion that may arise regarding which hygiene behaviors are adaptive vs those that are excessive; leading to difficulties in designing/performing exposure exercises and increasing the patient’s risk of contracting the virus. Instead of targeting contamination concerns head-on, clinicians should focus on behavioral activation and activity scheduling to keep patients preoccupied (eg, away from engaging in compulsive acts); strategies useful for managing symptoms of depression. Therapists in specialist centers may, in some cases, consider less efficacious techniques like imaginal exposure.^110^ Although these practical guidelines outlined in the report are sensible and useful given the unusual situation created by COVID-19, they are obviously not ideal in the long-term. Smartphone therapies aimed at targeting contamination fears and compulsive washing—once rigorously tested in randomized control trials (ie, has a clear evidence base)—may offer a more tolerable, cost-effective, and accessible alternative to ERP during times of pandemic. For instance, novel vicarious contamination and relief procedures (introduced above) may potentially provide avenues to conducting indirect exposures (without risking infection) and relieve the urge to engage in excessive washing.

## Summary and Future Directions

In this review, in addition to shedding light on symptoms in contamination-related OCD and reviewing current therapies, we discussed new technological approaches in light of global mental health and pandemics (COVID-19). As seen, there is currently an urgent need for novel, effective, and well-tolerated interventions for OCD, which can be adapted to these and other future changes in global circumstances. It is particularly important to design interventions that can target compulsive symptoms in the early stages of the disorder. Stimulus–response associations which may drive compulsions often become crystalized by the time patients typically receive a diagnosis and begin treatment. Notably, similar to other disorders with compulsive features (eg, addiction) OCD becomes harder to treat during later stages (eg, Gillan et al^85^ and do Rosario-Campos et al^114^).

As reviewed, a key limitation of existing psychological therapies for OCD (eg, ERP) includes limited tolerability/engagement for some patients, contributing to drop-outs and treatments being declined. There is thus a pressing need for gentler treatment options that do not require patients to touch highly anxiety-inducing objects (eg, indirect approaches). Tolerable, flexible technologically driven treatments are particularly needed during pandemics like COVID-19.

Future approaches should also address the limited accessibility of conventional treatment; for example, in light of global mental health and pandemics. They should overcome obstacles such as high cost, inconvenience of delivery, and geographical isolation. Smartphone interventions may help overcome limitations of conventional treatment, and potentially, provide a much-needed platform for treating contamination-related OCD during pandemic conditions. Despite these advantages, few smartphone apps are available for OCD; and those that do exist, have limited empirical evaluation thus far. Critically, these apps are based on ERP principles, which as noted do not suit all patients.

In sum, the great gap between symptom onset and treatment prolongs the chronicity of OCD,^115^ results in poorer treatment outcomes,^116^ and unnecessary suffering. Thus, to improve the chronicity, course and ultimately the high disease burden of OCD—taking into account global mental health obstacles and pandemics—it is critical to develop tolerable, accessible/transportable and cost-effective therapies. Novel smartphone therapies represent a promising avenue in terms of reducing this onset-to-treatment gap.
